# Evaluation of the United States Preventative Services Task Force Screening Guidelines for Breast Cancer in a Hispanic Underserved Population

**DOI:** 10.7759/cureus.8030

**Published:** 2020-05-08

**Authors:** Vivian Ngo, Mona Degan, Eugene Ho, David Lanum, Fanglong Dong, Michael Neeki

**Affiliations:** 1 Family Medicine, Arrowhead Regional Medical Center, Colton, USA; 2 Emergency Medicine, Arrowhead Regional Medical Center, Colton, USA

**Keywords:** breast cancer, mammograms, hispanic population, insurance

## Abstract

There are several major breast cancer guidelines that have been promoted by various health organizations. Arrowhead Regional Medical Center (ARMC) Family Health Centers adopted the current guideline by the U.S. Preventive Services Task Force (USPSTF), which recommends breast cancer screening with mammograms starting at age 50 for low-risk women. This study evaluates the effectiveness of this screening guideline in the selected Hispanic underserved population in San Bernardino, California (CA). This is a retrospective chart review study. Data were reviewed for any female with the confirmed diagnosis of breast cancer at the Family Health Centers between 2009 and 2018. The current study showed that 25% (40 of 160) of women diagnosed with breast cancer in this selected population were less than 50 years old. This finding suggests that high vigilance in breast cancer screening may be necessary in this population.

## Introduction

As of January 2016, there are more than 3.5 million women with a history of breast cancer in the U.S [1]. Female breast cancer is on the rise and the second-leading cause of cancer death in the U.S. The Centers for Disease Control and Prevention (CDC) reported that female breast cancer has the highest rate of new cancer cases [2].

Screening with mammograms is an effective method to detect breast cancer. According to the 2015 National Health Interview Survey, 50% of women 40 years of age and older reported having a mammogram within the past year and 64% reported having a mammogram in the past two years [3]. Among women 40 years of age and older, mammography prevalence increased from 29% in 1987 to 70% in 2000, but has since gradually declined [3].

Several major breast cancer guidelines have been promoted by various health organizations [4-10]. Although these health organizations agree that mammograms should be used for screening, the age and frequency of screening is debated. Most guidelines recommend that women with low risk start breast cancer screening at age 40. Before 2009, the U.S. Preventive Services Task Force (USPSTF) guidelines suggested screening starting at age 40 with yearly or biennially with mammograms. In 2009, these guidelines changed to screening at age 50. The recommendation is based on evidence that false-positive result and unnecessary testing is higher in age 40-49. The potential harm of biopsies, surgery, radiation, and chemotherapy outweighs the potential benefit. The American Academy of Family Physician agrees and supports the USPSTF guidelines [4]. Arrowhead Regional Medical Center (ARMC) Family Health Centers in San Bernardino, California (CA) also generally adopts these guidelines in practice.

The aim of this study is to evaluate the effectiveness of the USPSTF breast cancer screening guideline in a selected Hispanic underserved population in San Bernardino, CA, who seek care at the ARMC Family Health Centers. We hypothesize that this guideline will be inadequate for this patient population and may miss a significant percentage of breast cancer diagnoses.

## Materials and methods

The breast cancer guidelines promoted by various health organizations are summarised in Table 1.

**Table 1:** Different recommendations for breast cancer screening in women *Defined as women with a parent, sibling, or child with breast cancer **Defined as women with a parent, sibling, or child with a breast cancer gene (BRCA)1 or BRACA2 gene mutation ***Defined as women who test positive for BRCA1 or BRCA2 mutations, who are untested but have first-degree relatives with these mutations, or who are untested or negative for BRCA gene mutations but are estimated to have a lifetime risk of breast cancer of 20% or greater ****Defined as a genetics-based increased risk, with a calculated lifetime risk of 20% or more or a history of chest or mantle radiation therapy at a young age

	Recommendations for Breast Cancer Screening in Women
American Academy of Family Physicians 2016 [4]	Prior to age 50: Decision to start screening mammography should be an individual one Age 50-74: Biennial screening mammography Higher than average risk*: May benefit more than average-risk women from beginning screening in their 40s
American Cancer Society 2015 [7]	Age 40-44: Option to start screening with a mammogram every year Age 45-54: Mammograms every year Age 55 and older: Can switch to mammogram every other year or continue yearly mammogram, should continue as long as the woman is in good health and expected to live 10 years or longer Higher than average risk**: MRI and mammogram every year
American College of Obstetricians and Gynecologists 2011, 2017 [5,6]	Age 40-75: Should be offered screening mammography, should begin by no later than age 50, mammography every one to two years Above age 75: Decision to discontinue screening mammography should be based on shared decision process informed by woman’s health status and longevity Higher than average risk***: Twice-yearly clinical breast examinations, annual mammography, annual breast MRI, and breast self-examination
American College of Physicians [10]	Age 40-49: Discuss benefits and harms with women in good health, and order screening with mammography every 2 years if a women requests it Age 50-74 in good health: Encourage mammography every 2 years
American College of Radiology 2018 [8,9]	Age 40 and above: Annual mammographic screening, age to stop screening should be based on each woman’s health status rather than an age-based determination Higher risk****: Supplemental screening with contrast-enhanced breast MRI, may consider ultrasound if patient cannot undergo MRI
U.S. Preventative Services Task Force 2016 [4]	Prior to age 50: Decision to start screening mammography should be an individual one Age 50-74: Biennial screening mammography Higher than average risk*: May benefit more than average-risk women from beginning screening in their 40s

This is a retrospective study of patients who were cared for at the ARMC Family Health Centers with the confirmed diagnosis of breast cancer between 2009 and 2018 in San Bernardino, CA. San Bernardino is predominantly Hispanic; many are immigrants with language barriers and low socioeconomic status. The most common insurance plans in the population was Medicaid. Diagnosis was based on imaging and biopsy or on review of outside records from other institutions. Data were extracted from the medical record, including the age when they were diagnosed with breast cancer, insurance status, ethnicity, and self-reported family history of breast cancer. This study was approved by the Institutional Review Boards at ARMC.

## Results

A total of 160 female patients with a confirmed diagnosis of breast cancer were included in the final analysis. Among the 160 patients, the median age at the time of diagnosis was 56 (first quartile=49.5, third quartile=61) years, 50.6% (n=81) were Hispanic, 15% (n=24) patients had a family history of breast cancer, and 86.2% (n=89) had Medi-Cal/managed Medi-Cal insurance (Table 2).

Most notably, 25% (40 out of 160) of patients were diagnosed with breast cancer before age 50. In 2009 when the USPSTF guidelines changed, 66.7% (4 out 6) of patients were diagnosed before the age of 50. This percentage fell in the following years and shows a fluctuating trend (Table 3).

**Table 2:** The association between demographic variables and breast cancer diagnosis

	Breast cancer was diagnosed < 50 years	Breast cancer was diagnosed ≥ 50 years	Total number of breast cancer patients	P-value
Insurance				0.0045
Medicaid	37 (26.8%)	101 (73.2%)	138	
Medicare	0 (0%)	17 (100%)	17	
Private Insurance	3 (60%)	2 (40%)	5	
Ethnicity				0.0663
Caucasian	5 (12.8%)	34 (87.2%)	39	
African American	5 (17.2%)	24 (82.8%)	29	
Hispanic	27 (33.3%)	54 (66.7%)	81	
Other	3 (27.3%)	8 (72.7%)	11	
Family History of Breast Cancer				1
No	34 (25%)	102 (75%)	136	
Yes	6 (25%)	18 (75%)	24	

**Table 3:** Breakdown of breast cancer diagnosis by year (<50 vs 50+)

Year of diagnosis	Breast cancer was diagnosed < 50 years	Total number of breast cancer patients
2009	4 (66.7%)	6
2010	6 (46.2%)	13
2011	1 (12.5%)	8
2012	3 (25%)	12
2013	3 (15%)	20
2014	4 (19%)	21
2015	7 (35%)	20
2016	4 (14.3%)	28
2017	5 (38.5%)	13
2018	3 (15.8%)	19

## Discussion

The current study suggested that utilization of the USPSTF guidelines in our selected population may miss 25% of breast cancer diagnosis. Other studies have explored the age group of 39-49 and have noted that there is a statistically significant 15% reduction in breast cancer mortality for women randomly assigned to screening versus those assigned to controls [11]. The Age Trial specifically evaluated the effectiveness of screening women in their 40s from a community population in the United Kingdom and reported similar results [12].

Our results are consistent with the national average age of breast cancer diagnosis of 62 [1]. However, it is inconsistent with the CDC’s incidence rates by race and ethnicity. CDC reported that Caucasians had the highest incidence rate of breast cancer. The current study suggests that Hispanics had the highest incidence in our selected population. This might be associated with disparities such as low socioeconomic conditions, lack of insurance coverage, and lack of awareness. Women who have less than a high school education, who have no health insurance coverage, or who are recent immigrants to the U.S. are least likely to have had a recent mammogram [13]. The disparities make it important to establish screening guidelines that will not only help reduce the incidence of breast cancer but also the mortality, and subsequent emotional physical, familial, and emotional trauma. It will also help prevent over diagnosis and false-positive results that occur at a higher rate with annual screening which is in turn associated with fear and distress [13].

The goal of cancer screening is to detect malignancy early before complications and symptoms manifest. The hope is to diagnose and start treatment early to ensure effectiveness. Advances in breast cancer screening have led to early diagnosis and effective treatment. However, there are lower higher mortalities in certain groups. One study found that Hispanic women in the U.S. are less likely to be diagnosed at an early stage and approximately 20% more likely to more die from breast cancer compared to non-Hispanic White women [13]. With the advances in screening and treatment, there is overall improved survival in U.S. women with breast cancer. However, there is still a discrepancy between the social and racial groups.

This might be also associated with disparities such as low socioeconomic conditions, lack of insurance coverage, and lack of awareness. Women who have less than a high school education, who have no health insurance coverage, or who are recent immigrants to the U.S. are least likely to have had a recent mammogram [13]. The disparities make it important to establish screening guidelines that will not only help reduce the incidence of breast cancer but also the mortality, and subsequent emotional physical, familial, and emotional trauma [13].

Given this information, we seek to be more aware of our population’s disparities and aim to close the gap. One idea is increased patient awareness and education. One study found that participation in educational interventions by Spanish speaking educators improved breast cancer screening in a Hispanic population [14]. Cultural competency also may play a big role in increasing breast cancer screening rates. However, ultimately, it comes down to appropriate screening. Guidelines that are tailored to a specific population and demographic may also reduce false-negative results, which hinder diagnosis and treatment.

This study has several limitations. This is retrospective data and collection relied heavily on documentation, which may be insufficient or inaccurate. However, efforts have been applied to ensure a thorough chart review for maximum number of patients. Future studies may include patients from other primary care clinics in the area, emergency room patients, and breast surgery patients. Future research may include longitudinal follow up to assure that patients are properly monitored for prognosis and management.

## Conclusions

This study indicates that higher vigilance in the breast cancer screening may be necessary in our selected population. Our results emphasize the importance of disparity awareness. It also illustrates the need for family practitioners to be aware of their patient population and screen appropriately.
